# A Bloodless Method of Performing Tracheotomy

**Published:** 1878-07-20

**Authors:** Louis Henry


### A BLOODLESS METHOD OF PERFORMING TRACHEOTOMY.

We all know that the statistics in favor of tracheotomy, below the age of three, are not very favorable, some practitioners in Germany even refusing to perform the operation in croup or diphtheria, while some of the hospitals deny admission to the patients, as it increases the mortality percentage of their operative treatment to a very great extent. Out of 504 patients, on whom the operation of tracheotomy was performed in diphtheria, at Professor von Langenbeck's clinic during the last six years, 357, or 70.8 per cent., died. The causes of death were principally lobular pneumonia, croupous exudation, extending into the bronchi, asphyxia, exhaustion, paralysis of the laryngeal and pharyngeal muscles, and collapse.

The immediate danger and the sole cause of alarm to the inexperienced operator, in performing the usual operation of tracheotomy, is the bleeding. The operation I am about to describe, which may be considered entirely bloodless, is the one at present almost universally adopted in Germany when operating on children.

This operation of tracheotomy superior was first performed by Rose, Professor von Langenbeck's very able assistant, and is carried out in the following manner:

The little patient is slowly chloroformed, the mask being somewhat raised from the face if a paroxysm of coughing should set in. (I have constructed a chloroform apparatus, which may be regarded as an extensively modified Junker's inhaler, by which the amount of chloroform inhaled can be exactly regulated, and the whole apparatus worked with one hand, leaving the other hand free to feel the pulse, etc., and to assist the operator. This apparatus is in use at some of the Berlin hospitals.) As soon as the patient is chloroformed, a roller is thrust under the neck, and the head allowed to fall backward; this gives the front of the neck an arched appearance, and will throw the important parts into prominence. The operator now seeks the upper margin of the cricoid cartilage with the tips of his fingers, and makes a vertical incision through the skin exactly in the middle line of the neck, beginning about a small finger's width from above the upper margin of the cricoid, and extending about an inch and a half to two inches downward. The incised parts are drawn asunder, and the cricoid cartilage is thus so far exposed that, after steadying it with a finger, a transverse incision of not quite half an inch in length can be made as near its higher margin as possible. By this incision, the fascia, which envelopes the thyroid gland and conneets it with the trachea, is divided through. With a pincette, the operator now seizes hold of the lower border of this transverse incision, and, in the same way as the periosteum is severed off from the bone in a subperiosteal resection, he severs the fascia off from the trachea in a downward direction, either with a blunt hook or a director, pushing downward with the fascia all those veins which cause so many difficulties. As the operator gradually descends with the director, he unloosens the isthmus of the thyroid gland from the trachea, pushing the gland outward and downward, and lays the upper tracheal rings quite bare, so that they can now be seized and opened in the usual way.

This operation is particularly applicable to children, especially in those cases where immediate danger is apprehended, and the operation is to be performed at once. It is certainly preferable to the operation of tracheotomy inferior, which is performed below the thyroid gland, where the trachea lies much deeper and is covered by an extensive plexus of veins. In the operation just described, which is made above the isthmus of the thyroid gland, the number of cartilage rings that can be exposed and cut into will, of course, be more limited than in the inferior operation, and will also permit, should it be deemed necessary to enlarge the opening to extend the incision upward by dividing the cricoid cartilage, which, in children, none need hesitate to do.

Another great advantage in this operation is the fact that it does away with a staff of assistants. An intelligent nurse alone will be able to do all the assistance that is required. The incised parts can easily be kept asunder with a large, strong hair-pin, somewhat stretched to represent a large V, the free ends bent into half hooks, or two small hair-pins can be selected, the free ends bent and inserted under the incised parts on opposite sides, while the head of one pin is fastened to a piece of elastic, which passes round the back of the neck to the head of the other. This operation need but be practiced once or twice to insure confidence.—*Louis Henry, M.D., in British Med. Jour.*—(*Clinic.*)

